# Pressure measurement in the reticulum to detect different behaviors of healthy cows

**DOI:** 10.1371/journal.pone.0254410

**Published:** 2021-07-22

**Authors:** Josje Scheurwater, Miel Hostens, Mirjam Nielen, Hans Heesterbeek, Arend Schot, Rob van Hoeij, Hilde Aardema

**Affiliations:** 1 Department of Population Health Sciences, Faculty of Veterinary Medicine, University of Utrecht, Utrecht, The Netherlands; 2 Department of Clinical Sciences, Faculty of Veterinary Medicine, University of Utrecht, Utrecht, The Netherlands; 3 Brutton, Sint-Oedenrode, The Netherlands; University of Illinois, UNITED STATES

## Abstract

The aim of the current study was to investigate the relation between reticulorumen contractions and monitored cow behaviors. A purpose-built pressure measuring device was used and shown to be capable of detecting the known contraction patterns in the reticulorumen of four rumen-fistulated cows. Reticular pressure data was used to build a random forest algorithm, a learning algorithm based on a combination of decision trees, to detect rumination and other cow behaviors. In addition, we developed a peak-detection algorithm for rumination based on visual inspection of patterns in reticular pressure. Cow behaviors, differentiated in ruminating, eating, drinking, sleeping and ‘other’, as scored from video observation, were used to develop and test the algorithms. The results demonstrated that rumination of a cow can be detected by measuring pressure differences in the reticulum using either the random forest algorithm or the peak-detection algorithm. The random forest algorithm showed very robust performances for detecting rumination with an accuracy of 0.98, a sensitivity of 0.95 and a specificity of 0.99. The peak-detection algorithm could detect rumination robustly, with an accuracy of 0.92, a sensitivity of 0.97 and a specificity of 0.90. In addition, we provide proof of principle that a random forest algorithm can also detect eating, drinking and sleeping behavior from the same data with performances above 0.90 for all measures. The measurement device used in this study needed rumen-fistulated cows, but the results indicate that behavior detection using algorithms based on only measurements in the reticulum is feasible. This is promising as it may allow future wireless sensor techniques in the reticulum to continuously monitor a range of important behaviors of cows.

## Introduction

A properly contracting reticulorumen is important for digestion of a ruminant, a prerequisite for health and welfare of the animal [1]. The reticulorumen is responsible for mixing and breaking down digesta and is the site of microbial digestion of plant fiber. Much of the function was discovered already nearly 100 years ago using direct observation, palpation and measuring of pressure [2]. In the nineteen fifties and sixties, methods to measure pressure became more common, generally using rumen fistulated cows with balloons or fluid filled catheters [2–6]. Measuring normal and abnormal contraction patterns is key to understanding the digestive performance and important for possible future continuous monitoring of ruminant health and welfare. Here, we improve on existing methods to show that these patterns can be quantified with algorithms based on measurement of changing pressure in the reticulorumen and that these patterns link to specific observed behavior in cows (eating, rumination, drinking, sleeping).

**Fig 1:**
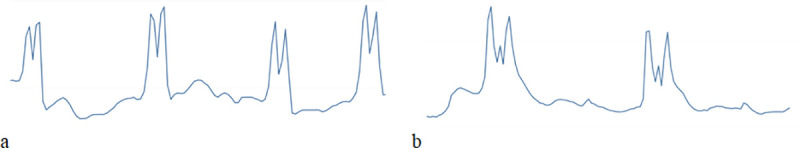
Reticular contractions during eating and rumination. Relative pressure differences illustrating two minutes of primary biphasic reticular contractions during eating (a), with a third contraction during rumination (b).

As described in [2, 7, 8], the contraction pattern of the reticulorumen consists of primary and secondary contraction cycles. The main function of the primary contraction cycle is to mix and transport ingesta back and forth between reticulum and rumen. The primary contraction cycle (A-wave) starts with a biphasic contraction of the reticulum followed by contractions of the different parts of the rumen in cranio-caudal direction. A third reticulum contraction occurs in the primary contraction cycle during rumination only (Fig 1). There is a secondary contraction cycle (B-wave) of the forestomach complex that is important for the eructation of gases without involvement of the reticulum and ruminal atrium [2, 7, 8]. We focus in this research on measurements of the A-wave in the reticulum.

Previous research based on relatively few observations already showed potential associations between the contraction cycles and behavior of the cow. For example, eating behavior has been linked with a shorter time interval between two contraction cycles [7–10], whereby the higher frequency of contractions may play a major role in increased outflow rate [10]. In contrast, stressed cows showed significantly longer intervals between contractions [8], while during periods of apparent sleep an absence of rumen motility was reported [7]. Furthermore, previous research showed contradictory results regarding the interval between contraction cycles during resting compared to rumination [7, 8, 10]. Comparing studies may be difficult because the state ‘resting’ is ambiguous. It is often defined as not-eating and not-ruminating but can still encompass a large variety of different behaviors, including actual resting.

Machine learning techniques can be valuable to link sensor data to animal behavior [11]. Algorithms based on Random Forests and Neural Networks were applied earlier in behavioral classification of cows fitted with sensors [12, 13]. A random forest (RF) is a learning algorithm to solve classification or regression problems. It does so by considering a collection of decision trees and combining different ‘trees’ into ‘forests’. An RF is defined as a combination of tree predictors such that each tree depends on the values of a random vector sampled independently and with the same distribution for all trees in the forest [14]. An RF performs well compared to other algorithms and is easier to use and more forgiving with regard to overfitting and outliers [1, 15]. Furthermore, RF are fast, flexible, and perform well even in the presence of a large number of features and a small number of observations [16]. The application of RF creates opportunities for the use of sensor data in behavior classification.

The aim of the current study was to investigate the relation between reticulorumen contractions, as measured by pressure differences in the reticulum, and specific monitored cow behavior (eating, rumination, drinking, sleeping). RF algorithms for detecting rumination and other behaviors was developed and evaluated. In addition, we developed a peak-detection algorithm for rumination based on visually inspection of differences in reticular pressure time series. We show that the RF algorithm based only on pressure differences in the reticulum can be used to detect several cow behaviors.

## Materials and methods

### Animals and animal observations

The study was performed from February 5, 2019 until April 15, 2019 in four rumen-fistulated Holstein Friesian cows at the Department of Farm Animal Health, Faculty of Veterinary Medicine, Utrecht University, Utrecht, the Netherlands. Animal procedures were approved by and in accordance with the guidelines of the Dutch Committee of Animal Experiments. The cows belonged to the same herd and had free-stall housing. Cows used for measurements were housed in a tie-stall barn, together with an accompanying cow, the evening before the day of data collection, to allow adaptation to the experimental environment. All rumen-fistulated cows were used to tie-stall housing. Before the start of each measurement period the health of the cow was clinically examined by a veterinarian. The animals were 7 to 9 years old; three were lactating and one was a dry cow. For further cow characteristics see Table 1. All animals were used to being handled by the animal observer.

**Table 1:** Characteristics of the four rumen-fistulated Holstein Friesian cows.

Animal	1	2	3	4
Days in milk	309	189	dried off before calving	320
Lactation no.	5	4	3	3
Milk yield (kg/d)^a^	19	32	-	24
Milk fat (%)^a^	5.00	5.01	-	4.52
Milk protein (%)^a^	3.73	3.35	-	3.41
Concentrate (kg DM)	0.9	4.5	0	1.8
Corn silage (kg DM)	4.0	4.0	2.5	4.0
Soya bean expeller (kg DM)	0.77	0.77	0	0.77
Formaldehyde treated soya bean expeller (kg DM)	0.61	0.61	0	0.61
Wilted grass silage (kg DM)	12.2	12.2	13.5	12.2

^a^ Milk yield, milk fat and milk protein based on the most recent test day before the start of the experiment.

During the experimental observation days, the cows were fed the same diet as they were used to in the free-stall housing and were milked twice daily. The diet consisted of 4 kg DM/day maize silage after morning and afternoon milking. Cows were fed according to Dutch standards [17]. A protein-rich supplement was supplied on top of the maize silage. Cows were provided with concentrates depending on milk production level. Dried-off cows received 2.5 kg DM/day maize silage in the evening. Fresh water and wilted grass silage were supplied ad libitum to all animals. See Table 1 for more details.

A video camera system recorded the cows during all measurements. The video data were analyzed by the same observer and cow behavior was scored every second to be either rumination, eating, drinking, sleeping or other (defined as none of the other behaviors). Sleeping was defined as lying immobile in a sleeping position, in a ventral recumbency with the head retroflexed to the flank. The video data analyzed by one human observer were used as the gold standard and were blindly scored independent of the pressure data, but there is always the chance of human error. In the process of data cleaning a second person checked a large part of the video data (JS) and did not observe any misinterpretations.

### Measurement device

**Fig 2:**
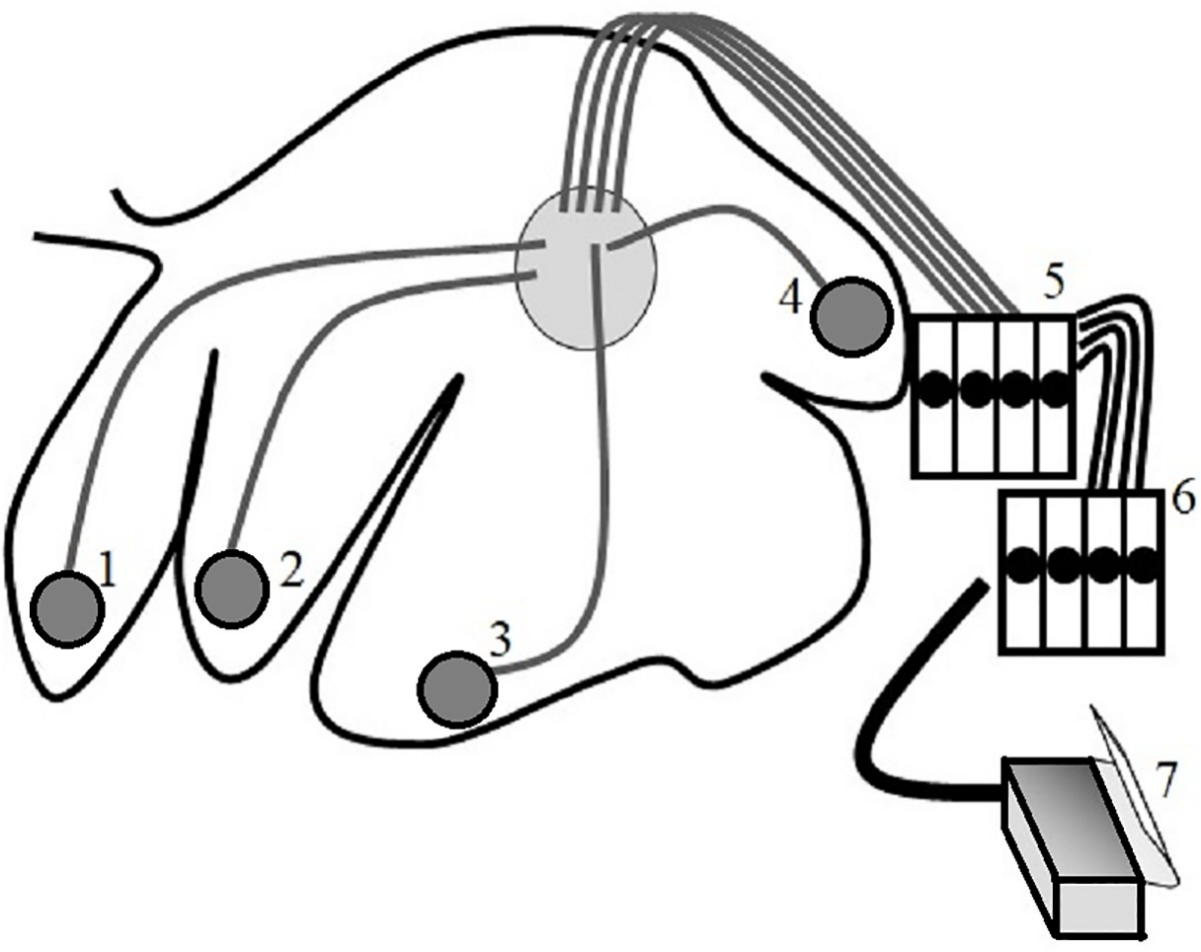
Schematic overview of the measurement device. (1) Reticulum (2) cranial sac of the rumen (3) ventral sac of the rumen (4) dorsal sac of the rumen (5) pressure transducers (6) amplifier (7) computer.

A purpose-built device to measure pressure differences in the reticulo-rumen was available. This device was originally developed for teaching (co-author AS) and is similar to approaches used in the literature that have previously been shown to measure pressure in the reticulorumen [4, 6, 18]. In this device, four water-filled open-tipped catheters are used to measure pressure differences in four different compartments of the reticulorumen, namely the reticulum, the cranial, the dorsal and the ventral sac of the rumen. Each catheter is connected to a pressure transducer. The transducer converts the water pressure to an electronic signal and sends it to an amplifier. The amplifier is connected to a computer to store and edit the data. A water-filled pressure bottle is connected to the transducer and a constant pressure from the bottle in the direction of the open tip of the catheter ensured that the catheter was continuously water-filled. To minimize fluctuations in the baseline pressure of the compartments, the position of the transducer can be adjusted to approximate the height of the catheter inside the reticulum at all times. It was possible to flush the catheter if clogged. See Fig 2 for a schematic overview of the device. The sensors measure pressure expressed in mV every 0.5 seconds. Using this device, data was collected during four hours at each measurement session between AM milking and PM milking. The proceedings did not visibly influence the rumen-fistulated cows in their normal behavior.

### Data analysis

Cow behavior, differentiated in ruminating, eating, drinking, sleeping and other, was scored from the video data and these observations were used to develop and test the algorithms based on reticulum pressure data. The pressure data were measured every 0.5 sec and matched to the behavior data based on the time in seconds, resulting in a dataset per 0.5 sec.

#### Random forest algorithm

In short, we first normalized the pressure data, after which the data was partitioned in windows of 120 sec with an overlap of 119.5 sec. The video data was matched with each 0.5 sec window. For each sliding window containing 240 pressure timepoints, features based on frequency were computed using three different methods. Fourier transform, power spectral density and autocorrelation were used to generate 30 low-dimensional features [19–21]. Based on these features, an RF classification model was developed to predict each behavior separately. The full code can be found at (https://doi.org/10.5281/zenodo.4538933).

Each separate behavior was predicted against a single class of all other behaviors, called the one-vs.-all technique [12]. The behaviors sleeping and drinking were not observed frequently during the study period, resulting in relatively few datapoints where a cow was either sleeping or drinking. When training the RF on these very unbalanced datasets (i.e., 5% in the positive class against 95% in the negative class), the one-vs.-all-algorithm is mainly trained to predict the majority class well and not the minority class. To achieve a better balance in the data for sleeping and drinking behavior, these minority classes were up-sampled three times in the data set with concomitant down-sampling of the majority class [16]. Through visual inspection of the AUC-curves numbers of parameters were chosen for each RF to limit overfitting. The RF model was trained with 70% of the data and tested using the remaining 30% (70/30 RF). For rumination, two additional approaches were conducted to validate the RF [22]:

1. The N-fold Stratified Cross Validation (SCV) approach. Data from all experimental days and different animals were combined and randomly split into five subsets. After that, one subset became a test set, and the remaining subsets were combined to become the training set. The process was repeated for each subset.
2. Leave-Out-One-Animal (LOOA) validation approach. Data from one animal became the test set and data from the remaining animals were combined to become the training set. Each animal became a test animal in turn.

#### Peak-detection algorithm

A peak-detection algorithm was developed based on patterns detected by visual inspection of the reticular time series data during rumination (Fig 1). The full algorithm can be found at (https://doi.org/10.5281/zenodo.4538933). The algorithm is aimed at detecting sharp peaks in the pressure landscape and then characterizes patterns in terms of sets of consecutive peaks that determine a contraction cycle, specifically assessing the duration of such sets and the timing between them. The peak-detection algorithm identifies sharp peaks in the pressure signal by determining for each increase and decrease whether, the change surpasses a pre-set threshold value within a certain timespan. To detect the distinct three-peak pattern of pressure differences during rumination, specific intermediate signal features such as highest peak, peak value, baseline and contraction interval were calculated using a sliding window of 75 seconds. The algorithm based on these features was used to characterize whether a cow was ruminating per 0.5s. Occasionally, only two peaks were observed in a contraction cycle. However, in those cases the time period between the two peaks had the same length as that of a regular three-peak pattern.

Visual inspection of pressure data time series did not show typical peak patterns for the other behaviors when compared to the video data, precluding the similar development of algorithms for behavior other than ruminating.

#### Random forest algorithm

In short, we first normalized the pressure data, after which the data was partitioned in windows of 120 sec with an overlap of 119.5 sec. The video data was matched with each 0.5 sec window. For each sliding window containing 240 pressure timepoints, features based on frequency were computed using three different methods. Fourier transform, power spectral density and autocorrelation were used to generate 30 low-dimensional features [19–21]. Based on these features, an RF classification model was developed to predict each behavior separately. The full code can be found at (https://doi.org/10.5281/zenodo.4538933).

Each separate behavior was predicted against a single class of all other behaviors, called the one-vs.-all technique [12]. The behaviors sleeping and drinking were not observed frequently during the study period, resulting in relatively few datapoints where a cow was either sleeping or drinking. When training the RF on these very unbalanced datasets (i.e., 5% in the positive class against 95% in the negative class), the one-vs.-all-algorithm is mainly trained to predict the majority class well and not the minority class. To achieve a better balance in the data for sleeping and drinking behavior, these minority classes were up-sampled three times in the data set with concomitant down-sampling of the majority class [16]. Through visual inspection of the AUC-curves numbers of parameters were chosen for each RF to limit overfitting. The RF model was trained with 70% of the data and tested using the remaining 30% (70/30 RF). For rumination, two additional approaches were conducted to validate the RF [22]:

3 The N-fold Stratified Cross Validation (SCV) approach. Data from all experimental days and different animals were combined and randomly split into five subsets. After that, one subset became a test set and the remaining subsets were combined to become the training set. The process was repeated for each subset.
4 Leave-Out-One-Animal (LOOA) validation approach. Data from one animal became the test set and data from the remaining animals were combined to become the training set. Each animal became a test animal in turn.

#### Analysis of algorithms

*The performance of the RF algorithm and the peak-detection algorithm was* evaluated using the video scores. Each datapoint of 0.5 seconds was scored by the algorithms to be positive or negative for a specific type of behavior. The video scores were used as the true behavior scores. A confusion matrix was constructed for each behavior type, counting true positives (TP), true negatives (TN), false positives (FP) and false negatives (FN) for all predictions by the algorithms. Based on this, the following standard metrics were calculated for each behavior:

Sensitivity (Se), is the fraction of video scores classified correctly by the algorithm as positive:

$$Se=\frac{TP}{TP+FN}$$ (1)

Specificity (Sp), is the fraction of video scores classified correctly by the algorithm as negative:

$$Sp=\frac{TN}{TN+FP}$$ (2)

Positive predictive value (PPV), is the fraction of algorithm scores classified as positive that are actually positive:

$$PPV=\frac{TP}{TP+FP}$$ (3)

Negative predictive value (NPV), is the fraction of algorithm scores classified as negative that are actually negative:

$$NPV=\frac{TN}{TN+FN}$$ (4)

The F1-score combines sensitivity and PPV, and provides a single fraction reflecting the ‘goodness’ of a classifier in the presence of rare classes:

$$F1=2*\frac{PPV*Se}{PPV+Se}$$ (5)

Accuracy (Acc) is the fraction of samples correctly classified by the algorithm:

$$Acc=\frac{TP+TN}{TP+TN+FP+FN}$$

We used the above formula to compute the classification performances of the classifiers for the different cow behaviors.

## Results

### Detection of contraction pattern

**Fig 3:**
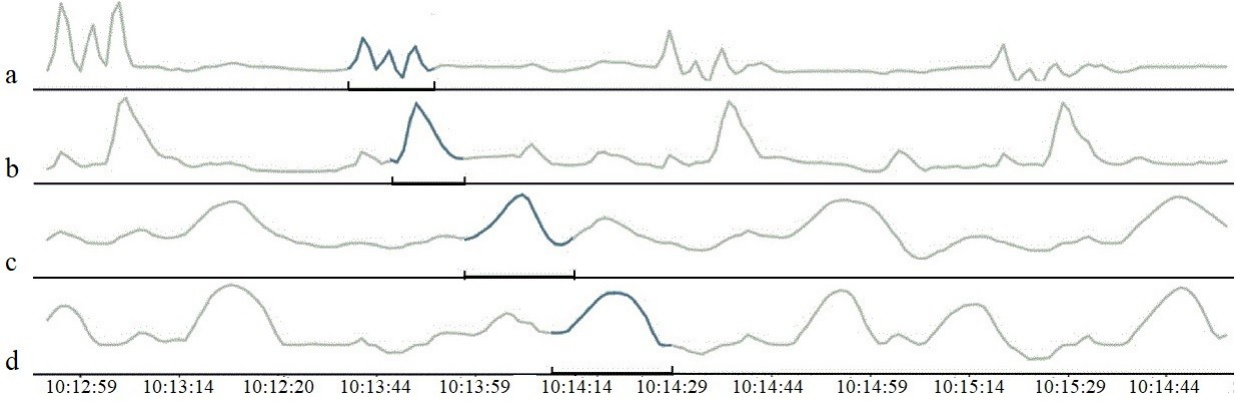
Pressure measured at four locations of the forestomach of a cow during rumination. (a) reticulum; (b) cranial ruminal sac; (c) dorsal blind sac; (d) ventral ruminal sac. The A-wave is highlighted in this figure and moves chronologically over the different compartments.

The contractions in four different compartments of the reticulorumen were detected and we were able to follow the A-wave over the reticulorumen (Fig 3). Each A-wave cycle could be detected in the reticulum first, followed by the cranial sac, the dorsal blind sac and finally the ventral sac of the rumen. A small pressure change was noted in the ventral ruminal sac at the moment of the contraction in the dorsal blind sac. This was likely due to noise caused by the measuring method.

### Descriptive aspects

In this experiment 57.5 hours of cow behavior data were analyzed resulting in 413,957 datapoints for the RF algorithm and 414,484 datapoints for the peak-detection algorithm. Four cows were used in this experiment. See Tables 2 and 3 for a summary of the number of observations per animal and per behavior type, respectively.

**Table 2:** Number of observations per cow.

Cow ID	Observations ^a^
2	109,288
13	72,564
21	97,337
84	135,295

^a^ Number of datapoints measured per 0.5sec.

**Table 3:** Number of observations per behavior.

Behavior	Observations ^a^
Rumination	132,828
Eating	106,915
Drinking	1,570
Sleeping	13,294
Other	159,877

^a^ Number of datapoints measured per 0.5sec.

**Fig 4:**
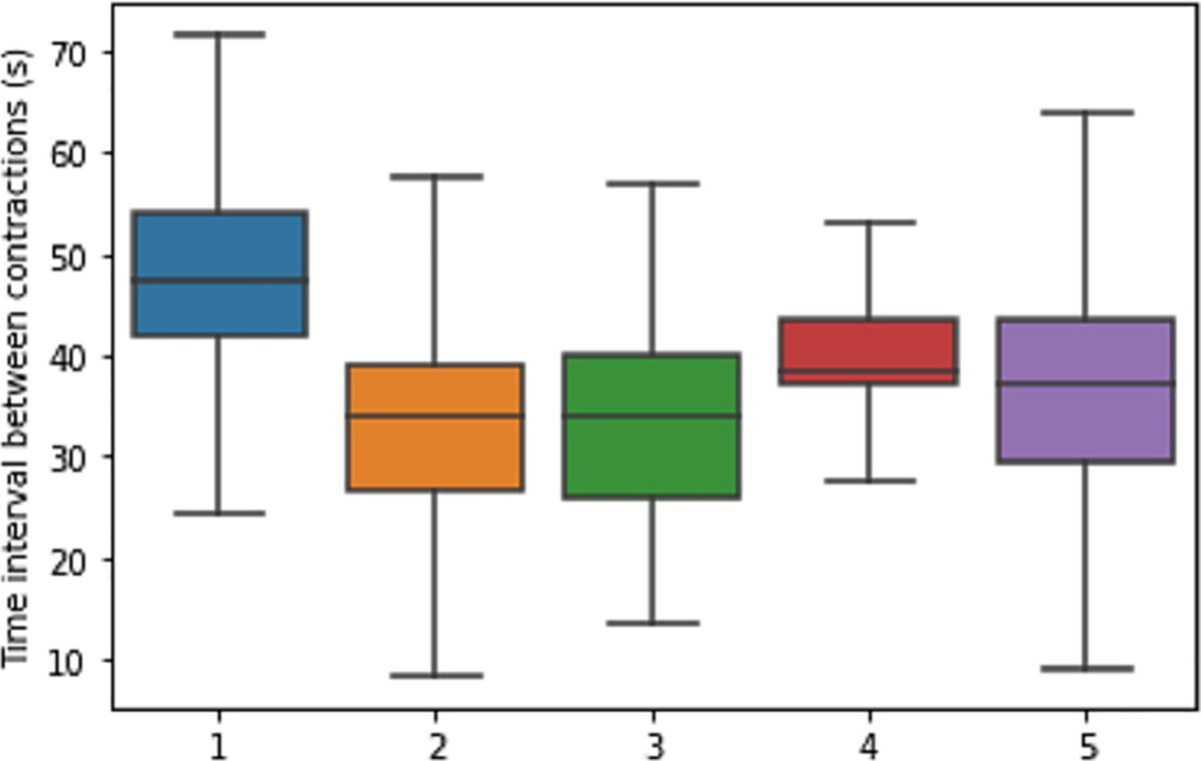
Boxplot of the time between two contraction cycles during the different behaviors. Time between two contraction cycles in seconds, during different behaviors: (1) Rumination; (2) Eating; (3) Drinking; (4) Sleeping; (5) Other. Boxplots depict the median and the upper and lower quartiles; whiskers depict quartiles ± 1.5 × the interquartile range (IQR); outliers not shown.

The average time between two contraction cycles per behavior type is given in Table 4; a difference in average time was found, with a wide overlap across behaviors. There was a significant difference (P < 0.001) of the average time between two contractions during rumination (48 sec) and eating (34 sec), as tested in a GLM model corrected for cow effect. However, even rumination and eating showed too much overlap to distinguish the two behaviors based on a cut-off value (Fig 4).

**Table 4:** Time in seconds between two contraction cycles during different cow behaviors.

Behavior	Mean (SD)	Median (IQR)
Rumination	48 (12.1)	48 (12.5)
Eating	34 (12.7)	34 (13.0)
Drinking	35 (12.9)	34 (14.4)
Sleeping	41 (11.4)	39 (6.5)
Other	40 (23.1)	37 (14.5)

### Detection of rumination

Rumination could also be detected with an RF algorithm trained on 70% of the dataset and tested on the other 30%. The RF algorithms showed very robust performances for detecting rumination, and also when repeated five times with a 5-fold stratified cross validation technique. All five RFs for rumination showed the same accuracy of 0.98, sensitivity of 0.95 and specificity of 0.99. In contrast, when the RF was trained on data of three cows and tested on the data of the other cow, in the LOOA validation the sensitivity and accuracy were lower and also the other metrics were more variable (see Table 5), possibly due to one aberrant animal with extreme low sensitivity of 0.56. The peak-detection algorithm could detect rumination with an accuracy of 0.92 a sensitivity of 0.97 and a specificity of 0.90. For other extracted metrics see Table 5.

**Table 5:** Performance of algorithms for rumination.

Algorithm	Se	Sp	PPV	NPV	F1	Acc
Peak-detection	0.97	0.90	0.82	0.98	0.89	0.92
RF 70/30	0.94	0.99	0.98	0.97	0.96	0.98
RF SCV ^a^	0.95 (0.002)	0.99 (0.000)	0.99 (0.001)	0.98 (0.001)	0.97 (0.002)	0.98 (0.001)
RF LOOA ^b^	0.73 (0.105)	0.96 (0.019)	0.89 (0.067)	0.88 (0.076)	0.80 (0.057)	0.88 (0.049)

RF, Random Forest; SCV, Five-fold Stratified Cross Validation; LOOA, Leave-out-one-animal based on four cows.

^a^ Average and standard variation over 5 folds.

^b^ Average and standard deviation over four cows.

### Proof of principle for detection of multiple behaviors

Four mutually exclusive behavior classes were scored during the experiment: Rumination, Eating, Drinking and Sleeping. Table 6 illustrates the performance metrics of the one-vs.-all RF algorithms (RF 70/30) of these behaviors. Sleeping and Drinking had a small representation, resulting in unbalanced datasets that were resampled. Our data suggest that also these cow behaviors can be detected by measuring pressure differences in the reticulum.

**Table 6:** Random forest algorithms (RF 70/30) for different behaviors.

Behavior	TP	FP	TN	FN	Se	Sp	PPV	NPV	F1	Acc
Rumination	37,410	570	83,940	2,196	0.94	0.99	0.98	0.97	0.96	0.98
Eating	29,231	903	91,006	2,976	0.91	0.99	0.97	0.97	0.94	0.97
Drinking ^a^	467	17	454	4	0.99	0.96	0.96	0.99	0.98	0.98
Sleeping ^a^	3,947	240	3,749	42	0.99	0.94	0.94	0.99	0.97	0.96

^a^ Numbers reflect the resampled dataset to balance the data.

## Discussion

The measuring device used in this study indeed detected the expected contraction pattern in the reticulorumen of the rumen-fistulated cows [2, 7, 8]. The results demonstrate that rumination activity of a cow can be detected by measuring pressure differences in the reticulum using either an RF algorithm or an algorithm based on visual inspection of reticulorumen pressure time series. A proof of principle is presented indicating that an RF algorithm can in addition detect eating, drinking and sleeping from the same data. These results are promising as they may allow future wireless pressure sensors in the reticulum to continuously monitor a range of important behaviors of cows.

Most previous studies that differentiated eating, rumination and resting based detection on the mean time interval between contraction cycles. We found that the time between two contraction cycles is shortest during eating, similar to other studies [7–10], but that the large variation around the means precluded setting a clear threshold to separate eating from other behavior. In our study, the time interval between two contractions was defined as the time with no peaks. This is in contrast to the other studies where the time interval was the time between the last peak top of one contraction and the top of the first peak of the next contraction. Therefore, the exact time intervals in seconds were not comparable across studies. Furthermore, we did not find an absence of rumen motility during periods of apparent sleep as detected by others [7]. However, in our data there is no certainty of sleep because we scored sleeping based on direct observation and not on brain activity [23].

The RF algorithm was able to differentiate between peaks and noise when trained to detect rumination, shown by a high specificity (99%). Likewise, the RF for eating had a high specificity (99%), indicating that the RF approach is also able to distinguish eating from other behaviors. Inspection of the features used by the RF for rumination and the RF for eating showed a clear difference in which features were important (https://doi.org/10.5281/zenodo.4538933). The different features used in the two RF’s demonstrate that both behaviors have their own distinguishable pressure pattern. Studying the feature sets could provide additional insight into the differences in contraction pattern. The Stratified Cross Validation showed minimal variance in the performances, suggesting that the RF algorithm is robust. The weaker performance of the Leave-Out-One-Animal approach is possibly caused by the data of one of the four cows. This particular cow was the only dry cow in this experiment. Whether the dry period of the cow contributed to the difference needs more investigation. Potential causes that may account for the observed differences in reticulum contraction patterns compared to lactating cows, may be the diet and consequently the content of the reticulorumen, volume, or abdominal pressure by a pregnancy. Another reason could be that the reticular contraction patterns show important variability between individual cows and that the RF algorithm should be developed on more than three animals, or that more finetuning and filtering of the sensor data is necessary to allow comparison of different animals.

The peak detection algorithm showed that it was possible to distinguish between ruminating or not-ruminating with one algorithm independent of the specific animal. The algorithm functioned well to detect rumination for the complete dataset including data of all four tested animals. Other behaviors could not be detected by an algorithm based on visual inspection of patterns in the data. The specificity of the peak-detection algorithm for rumination was only 90% compared to a sensitivity of 97%. The reason for the relatively low specificity could be that the data was not normalized, resulting in noise being classified falsely as rumination peaks.

Drinking and sleeping could be detected with an RF algorithm with more than 90% sensitivity and specificity. Without the use of EEG techniques, the posture of the cow currently appears to be the most reliable way to score sleeping behavior. Sufficient sleep time is important for the welfare of cows and essential for both an adequate metabolic system and the immune function. However, little is known about sleep in cattle [23]. Even though the datasets for sleeping and drinking were only small it still suggests that reticulum pressure could probably be used with an RF algorithm to detect other cow behaviors than rumination and eating.

In this study we presented a proof of principle for detecting different behaviors of cows by measuring pressure in the reticulum. The results suggest that with even standard default RF methods important behaviors could be detected. The behavioral types in this study were chosen because they have no overlap in the sense that no two of these behaviors are generally observed in the same individual simultaneously. For this proof of principle, the aim was to explore the relation between reticular pressure patterns and different cow behaviors, not to be able to predict what a cow was doing based on pressure patterns in the reticulum. If in further studies the multiple behaviors need to be classified at a particular moment, the corresponding set of binary classifiers can be combined by either ensemble methods or a multiclass algorithm. In addition, the behavior types in this study were chosen because they can be scored as approximately binary (presence/absence) and are therefore well-suited for the approach. Because behavior is generally not binary, future algorithms might improve by utilizing multiclass algorithms.

In this study the peak-detection algorithm was developed based on visually observed patterns of pressure differences, while, machine learning RF’s are based on automatic extraction of features. With machine learning techniques like RF, patterns not apparent by visual inspection can be detected using higher order features. For example, the pressure data is transformed from a time domain to a frequency domain by Fourier Transformation. While this allows for the detection of more subtle and higher-order patterns, the abstract RF-approach complicates mechanistic interpretation of the patterns that are discovered.

It was not our aim to draw conclusions on the behavior of healthy cows or on biological mechanisms underlying different types of behavior. The aim was proof of principle for the monitoring of behavior. There are limitations to the study and caution should therefore be taken before interpreting our results in a wider context beyond this proof of principle. First, the number of animals in our study was small. Second, it is unclear in what way pressure patterns of the rumen-fistulated cows, even if these animals are in all other aspects ‘healthy’, can be taken as representative of a normally functioning cow. Furthermore, the fistula can also influence measurements in our set-up. The method itself may therefore limit biological interpretation of the patterns we were able to quantify. In addition, the algorithms, whether RF or informed by visual inspection do not immediately allow mechanistic interpretation of the patterns that are observed in terms of biological processes involved in different types of behavior. This is less of a problem for monitoring when one has such high specificity and sensitivity as in our study, but clues to understanding the underlying biology are a more difficult challenge.

The future perspective is that behavior detection based on measurement of only the A-wave in the reticulum is feasible and would therefore be relevant for wireless sensor techniques. Wireless ruminal sensors are available in commercial dairy farms and particularly in animal research [24–26]. Most orally applied sensors migrate towards the reticulum and can measure temperature or pH in the reticulum continuously [26]. Measuring rumen motility by three-axis acceleration data using a sensor bolus has also been explored [9]. In another study, actual pressure differences are measured by a bolus every 10 sec [27], which may not yet be frequent enough to monitor reticulorumen contraction waves sufficiently accurately for the identification of cow behavior.

Measuring reticulorumen contractions is not only interesting for detection of different cow behaviors, but also for identifying cows with welfare problems or at risk for disease [1, 9]. For example, until now there is no valid method to score sleeping behavior, which is important for cow welfare. The use of reticular pressure differences may be an important parameter as the basis for an index of sleeping behavior in the near future. In addition, the frequency and amplitude of reticulorumen contractions are negatively influenced by many factors including metabolic diseases, anorexia and other diseases that cause pain or fever [9]. The pH in the reticulorumen can also influence the reticulorumen contractions and acidosis has been shown to reduce the frequency of ruminal contractions, resulting in reticulorumen stasis [4]. Reticulorumen stasis may lead to diseases such as displaced abomasum and ruminal tympany [9]. Therefore, reticulorumen motility is an important measurable characteristic to identify cows at risk for disease [1].

## Conclusions

Our results strongly suggest that measurements of pressure differences in the reticulum can be utilized to detect various behaviors of cows based on RF algorithms. Our data provided a proof of principle for future automatic monitoring of ruminating, eating, drinking and sleeping behavior of cows.

## References

[1] Silva de Tarso SG da. The Rumen as a Health Thermometer: Importance of Ruminal Function to the Metabolic Balance in Ruminants–Mini Review. J Dairy, Vet Anim Res 2017;5. 10.15406/jdvar.2017.05.00139.

[2] Sellers AF, Stevens CE. Motor functions of the ruminant forestomach. Physiol Rev 1966;46:634–61. doi: 10.1152/physrev.1966.46.4.634 5341713

[3] Balch CC, Balch DA, Johnson VW, Turner J. Factors Affecting the Utilization of Food by Dairy Cows. Br J Nutr 1953;7:212–24. doi: 10.1079/bjn19530026 13081935

[4] Egert-McLean AM, Sama MP, Klotz JL, McLeod KR, Kristensen NB, Harmon DL. Automated system for characterizing short-term feeding behavior and real-time forestomach motility in cattle. Comput Electron Agric 2019;167:105037. 10.1016/j.compag.2019.105037.

[5] Okine EK, Mathison GW, Hardin RT. Effects of changes in frequency of reticular contractions on fluid and particulate passage rates in cattle. J Anim Sci 1989;67:3388–96. doi: 10.2527/jas1989.67123388x 2613584

[6] Quigley J. P.; Brody DA. A physiologic and clinical consideration of the pressures developed in the digestive tract. Am J Med 1952;13:73–81. doi: 10.1016/0002-9343(52)90082-x 12976409

[7] Church DC. Motility of the gastro-intestinal tract. Dig. Physiol. Nutr. Ruminants, 1976, p. 69–98.

[8] Braun U, Rauch S. Ultrasonographic evaluation of reticular motility during rest, eating, rumination and stress in 30 healthy cows. Vet Rec 2008;163:571–4. doi: 10.1136/vr.163.19.571 18997187

[9] Arai S, Okada H, Sawada H, Takahashi Y, Kimura K, Itoh T. Evaluation of ruminal motility in cattle by a bolus-type wireless sensor. J Vet Med Sci 2019;81:1835–41. doi: 10.1292/jvms.19-0487 31685723 PMC6943329

[10] Okine EK, Mathison GW, Kaske M, Kennelly JJ, Christopherson RJ. Current understanding of the role of the reticulum and reticulo-omasal orifice in the control of digesta passage from the ruminoreticulum of sheep and cattle. Can J Anim Sci 1998;78:15–21. 10.4141/A97-021.

[11] Neethirajan S. The role of sensors, big data and machine learning in modern animal farming. Sens Bio-Sensing Res 2020;29:100367. 10.1016/j.sbsr.2020.100367.

[12] Smith D, Rahman A, Bishop-Hurley GJ, Hills J, Shahriar S, Henry D, et al. Behavior classification of cows fitted with motion collars: Decomposing multi-class classification into a set of binary problems. Comput Electron Agric 2016;131:40–50. 10.1016/j.compag.2016.10.006.

[13] Vázquez Diosdado JA, Barker ZE, Hodges HR, Amory JR, Croft DP, Bell NJ, et al. Classification of behaviour in housed dairy cows using an accelerometer-based activity monitoring system. Anim Biotelemetry 2015;3:1–14. 10.1186/s40317-015-0045-8.

[14] Breiman L. Random forests. Mach Learn 2001;45:5–32.

[15] Horning N. Random Forests: An algorithm for image classification and generation of continuous fields data sets. Proc. Int. Conf. Geoinformatics Spat. Infrastruct. Dev. Earth Allied Sci. Osaka, Japan, vol. 911, 2010.

[16] Ziegler A, König IR. Mining data with random forests: Current options for real-world applications. Wiley Interdiscip Rev Data Min Knowl Discov 2014;4:55–63. 10.1002/widm.1114.

[17] Veevoederbureau C. Tabellenboek Veevoeding. Voedernormen Rundvee, Schapen En Geiten, En Voederwaarden Voermiddelen Herkauwers (in Dutch) Hague, Netherlands 2016.

[18] Okine EK, Tesfaye A, Mathison GW. Relationships between reticular contractions and digesta passage in steers consuming alfalfa hay and barley straw combinations ad libitum. J Anim Sci 1993;71:3043–51. doi: 10.2527/1993.71113043x 8270526

[19] Bracewell RN. The Fourier transform and its applications. vol. 31999. McGraw-Hill New York; 1986.

[20] Kaur M, Singh B, Seema. Comparison of different approaches for removal of Baseline wander from ECG signal. Int Conf Work Emerg Trends Technol 2011, ICWET 2011—Conf Proc 2011:1290–4. 10.1145/1980022.1980307.

[21] Parák J, Havlík J. ECG signal processing and heart rate frequency detection methods. Proc Tech Comput Prague 2011.

[22] Rahman A, Smith D V., Little B, Ingham AB, Greenwood PL, Bishop-Hurley GJ. Cattle behaviour classification from collar, halter, and ear tag sensors. Inf Process Agric 2018;5:124–33. 10.1016/j.inpa.2017.10.001.

[23] Ternman E, Hänninen L, Pastell M, Agenäs S, Nielsen PP. Sleep in dairy cows recorded with a non-invasive EEG technique. Appl Anim Behav Sci 2012;140:25–32. 10.1016/j.applanim.2012.05.005.

[24] Falk M, Münger A, Dohme-Meier F. Technical note: A comparison of reticular and ruminal pH monitored continuously with 2 measurement systems at different weeks of early lactation. J Dairy Sci 2016;99:1951–5. doi: 10.3168/jds.2015-9725 26723129

[25] Denwood MJ, Kleen JL, Jensen DB, Jonsson NN. Describing temporal variation in reticuloruminal pH using continuous monitoring data. J Dairy Sci 2018;101:233–45. doi: 10.3168/jds.2017-12828 29055552

[26] Dijkstra J, Van Gastelen S, Dieho K, Nichols K, Bannink A. Review: Rumen sensors: Data and interpretation for key rumen metabolic processes. Animal 2020;14:S176–86. doi: 10.1017/S1751731119003112 32024561

[27] Kilic U. Use of wireless rumen sensors in ruminant nutrition research. Asian J Anim Sci 2011;5:46–55.

